# Photo‐switchable Fluorescence in Hydrogen‐Bonded Liquid Crystals

**DOI:** 10.1002/chem.202001696

**Published:** 2020-09-17

**Authors:** Alexander Kappelt, Michael Giese

**Affiliations:** ^1^ Organic Chemistry University of Duisburg-Essen Universitätsstrasse 7 45117 Essen Germany

**Keywords:** fluorescence, hydrogen-bonded liquid crystals, photo switching, responsive materials, stilbazoles

## Abstract

A series of hydrogen‐bonded liquid crystals showing switchable fluorescence is reported. The fluorescence behavior results from the unique combination of hydrogen bonding, liquid crystallinity, and photobasicity. Thus, the molecular mobility in the mesophase is essential for the reversible photo‐initiated proton transfer switching on the fluorescence of the assemblies. The application potential of the materials for photo‐patterning was demonstrated.

## Graphical abstract

**Turn the light on**: Supramolecular materials with reversible photo‐switchable fluorescence were obtained by combining 4‐alkoxystilbazoles with hydroxy benzoic acids. Using the photobasicity/acidity of the assemblies lead to fluorescent materials upon irradiation in the mesophase

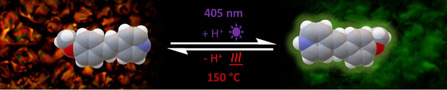

Organic photoluminescent materials with stimuli‐responsive properties have recently gained much attention due to their potential for application in, for example, biological sensing,^[1]^ light‐emitting diodes,^[2]^ logic gates,^[3]^ and anti‐counterfeiting.^[4]^ In addition, gaining control over the luminescence by external stimuli, for example, by temperature or irradiation with light is highly valuable, as it provides access to responsive functional materials. With respect to the design of such luminescent materials, a self‐assembly approach based on noncovalent bonds provides many advantages as it facilitates the fabrication, processing and recycling of materials.^[5]^ The functional materials are obtained by simple mixing of pre‐tailored building blocks in an appropriate solvent at room temperature. Furthermore, the dynamic nature of noncovalent interactions provides access to materials that respond reversibly to external stimuli or damages (self‐healing/‐repair).^[6]^

In the late 1980s, Kato,^[7]^ Fréchet,[^7b^, ^8^] and Lehn^[9]^ introduced a new design concept for liquid crystals by self‐assembly of benzoic acid derivatives or based on the complementarity of between benzoic acid groups and pyridyl derivatives.[^6a^, ^6b^, ^10^] Later, Bruce and co‐workers demonstrated that phenols are also suitable proton donors for the formation of hydrogen‐bonded assemblies with liquid‐crystalline properties.^[11]^ Although many of these examples employ substituted stilbazole derivatives, only little is reported about the luminescence behavior.^[12]^

In 2016, we introduced a modular approach for systematic studies on the design of hydrogen‐bonded liquid crystals with tailor‐made properties.[^13^, ^14^, ^15^] The initial studies have granted insights into the structure–property relationships of the assemblies, for example, the impact of the substitution pattern of the hydrogen‐bond donors,^[16]^ the fluorination degree,^[13b]^ and the linking group in the hydrogen‐bond accepting moieties was systematically investigated.^[14]^ A fundamental understanding of the structure–property relationship is crucial for the development of new functional materials. For instance, our findings helped us to stabilize liquid‐crystalline blue phases (BP I)^[15]^ or to manipulate the optical properties of photonic crystals by infiltration of the porous materials with hydrogen‐bonded liquid crystals.^[17]^ Recently, we used the modular methodology to create luminescent liquid crystals through their aggregation‐induced emission behavior.^[18]^

The present work employs the modular concept to yield photo‐switchable, luminescent materials. Therefore, a series of hydroxybenzoic acids (4‐ (**4HBA**), 3‐ (**3HBA**) and 2‐hydroxybenzoic acid (**2HBA**); Scheme 1) were combined with alkoxy‐stilbazole (**St8**) to form hydrogen‐bonded assemblies with liquid‐crystalline properties. The modularity of our approach allows efficient fine‐tuning of the hydrogen‐bonding capability and the acid–base equilibrium to obtain assemblies with balanced intermolecular forces enabling photoswitchable fluorescence behavior.

**Scheme 1:**
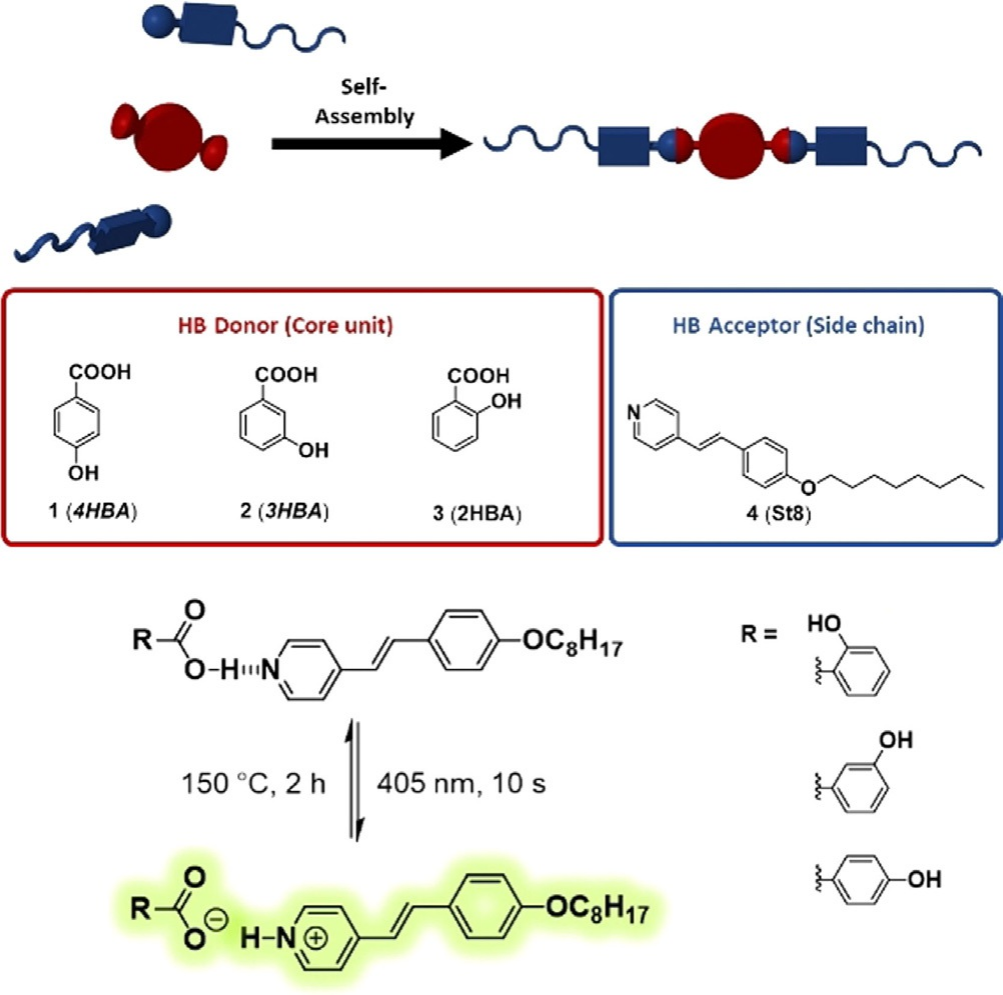
Schematic representation of the modular approach yielding photoswitchable fluorescent assemblies.

In total, six hydrogen‐bonded liquid crystals were obtained by mixing solutions of the HBAs with **St8** in 1:1 and 1:2 ratios in acetone. After removal of the solvent under reduced pressure, the materials were obtained and analyzed with respect to their liquid‐crystalline behavior. The formation of the hydrogen‐bonded assemblies was proven by FTIR spectroscopy (for details see the Supporting Information). Taking the **4HBA**‐based assemblies as representative examples, the OH signal shifts from 3364 to 3116 cm^−1^ for the 1:1 mixture and disappears for the 1:2 mixture. In addition, the C=O band undergoes a shift from 1669 to 1656 cm^−1^ for the 1:1 and to 1687 cm^−1^ for the 1:2 complex. An additional proof of the formation of the assemblies is given by the occurrence of liquid‐crystalline properties of the assemblies. None of the starting materials showed mesomorphic properties upon self‐assembly and formation of the hydrogen‐bonded materials; however, liquid crystallinity was observed.

In the following discussion, we will focus on the 1:2 assemblies because they performed better with respect to liquid crystallinity and photo‐switchable fluorescence. These assemblies showed enantiotropic liquid‐crystalline behavior. For the **3HBA**(**St8**)_**2**_ and **4HBA**(**St8**)_**2**_ assemblies, the characteristic Schlieren texture of a nematic phase was observed under a polarized optical light microscope (POM; Figure 1). The **2HBA**(**St8**)_**2**_ assemblies, however, tend to form focal‐conic textures indicative for a smectic phase. We attribute the change in the nature of the mesophase to the formation of 1:1 assembly, as one of the OH groups is blocked by intramolecular hydrogen bonding to the carboxylic group.^[19]^ This is in line with the observed inhomogeneous melting/cooling behavior and the obtained IR data. The POM results were supported by differential scanning calorimetry (DSC, for details see the Supporting Information). The temperature range of the mesophases decreased in the order **3HBA**>**2HBA**>**4HBA** from Δ*T*=70.2 to 54.3 and to 44.3 °C, respectively, upon cooling.

**Figure 1:**
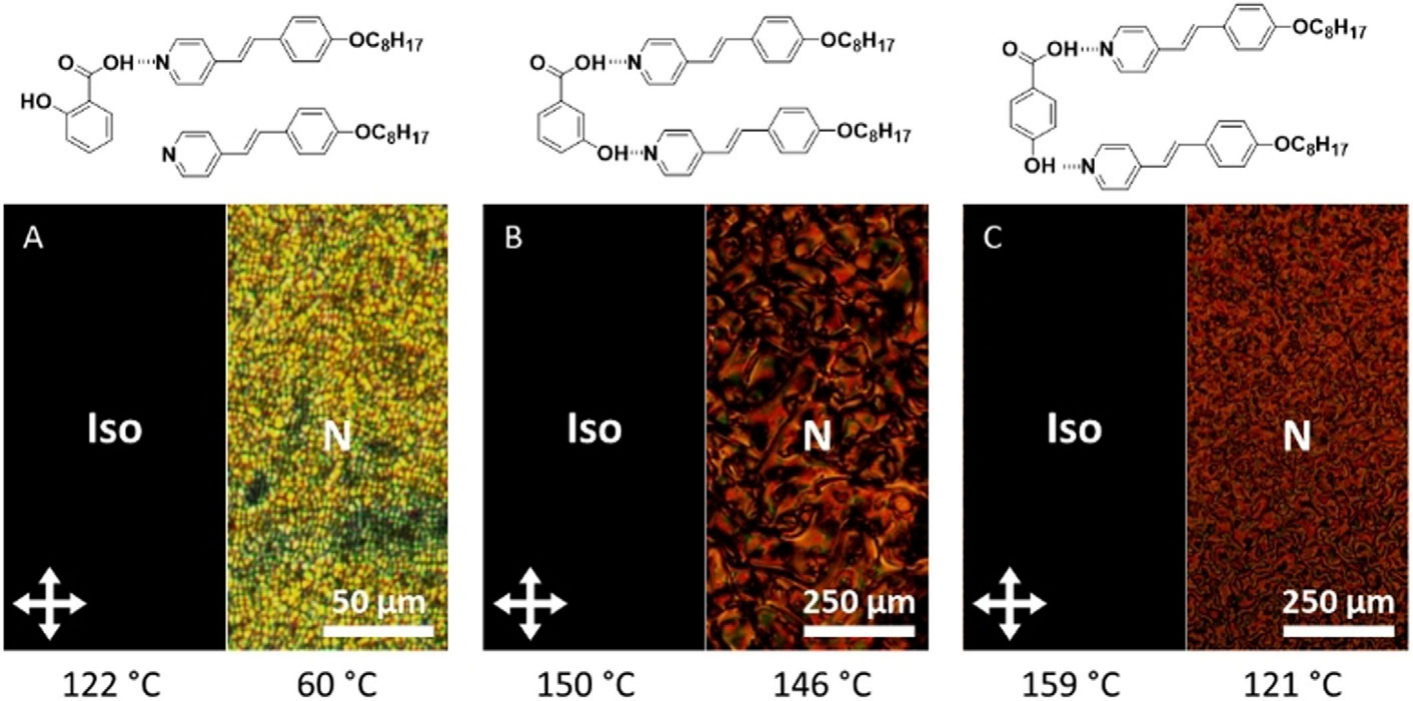
POM images of A) **2HBA**(**St8**)_**2**_ at 122 (left) and 60 °C (right), B) **3HBA**(**St8**)_**2**_ at 150 (left) and 146 °C (right) and C) **4HBA**(**St8**)_**2**_ at 159 (left) and 121 °C (right).

As already mentioned, the most interesting property of the obtained assemblies is their photoswitchable fluorescence. The properties of supramolecular functional entities rely on a well‐orchestrated interplay of various intermolecular forces. Subtle changes in the molecular structure can result in tremendous differences in the properties of the functional assemblies. In the present series, the substitution pattern of the hydrogen bond‐donating HBAs is varied; this has an impact on the shape of the resulting assemblies. Moreover, the acidity of the HBAs varies, and this controls the strength of the noncovalent bonds and the protonation state in the assemblies.

After removal of the acetone, the 1:2 assemblies were obtained as colorless or slightly yellow crystalline solids. No visible fluorescence was observed under UV light. However, after one heating/cooling cycle, the assemblies differed in their fluorescence behavior. The **2HBA**(**St8**)_2_ assembly showed visible blue light fluorescence, whereas **3HBA**(**St8**)_2_ and **4HBA**(**St8**)_2_ appeared only slightly fluorescent (Figure 2). The occurrence of the fluorescence can be attributed to the intermolecular proton transfer between the HBAs and the stilbazole, which is supported by IR investigations (Figure S4 in the Supporting Information). The differences in the fluorescence behavior indicates the crucial impact of the substitution pattern in the hydrogen bond donating units and can be attributed to the differences in the acidity of the HBAs. The acidity of the hydrogen bond donating moieties decreases in the order **2HBA** (p*K*_a_=2.75)>**3HBA** (p*K*_a_=3.90)>**4HBA** (p*K*_a_=4.61).^[20]^ Thus the protonation equilibrium in the **2HBA**(**St8**)_**2**_ is shifted to the protonated stilbazole and the deprotonated **2HBA**, causing the fluorescence.^[21]^ In contrast, the assemblies based on **3HBA** and **4HBA** appear non‐ or only slightly fluorescent. However, bringing these assemblies into their mesophases, followed by irradiation with light of *λ*=405 nm turns on the fluorescence; this can be attributed to a photo‐initiated proton transfer. Doty et al. reported that the photo‐excitation of *trans*‐methoxy stilbazole yields a p*K*_a_ shift from 4.93 in the ground state to 13.02 in the excited state. The strong increase in basicity shifts the equilibria significantly to the protonated, fluorescent stilbazole species.^[22]^

**Figure 2:**
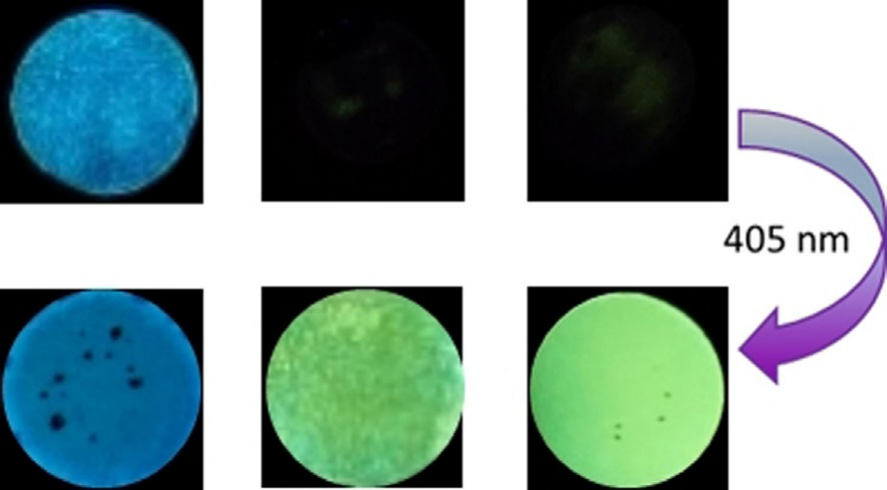
1:2 Mixtures of **2HBA**(**St8**)_**2**_ (left), **3HBA**(**St8**)_**2**_ (center) and **4HBA**(**St8**)_**2**_ (right) under UV light; upper row: untreated; lower row: irradiated with 405 nm light at 130 °C for 15 s.

In order to evaluate the fluorescence behavior, spectra of the assemblies in their fluorescent state were recorded. Whereas **2HBA**(**St8**)_**2**_ emits at *λ*_em_=463 nm (*λ*_ex_=428 nm), the **3HBA**(**St8**)_**2**_ and **4HBA**(**St8**)_**2**_ assemblies emit at *λ*_em_=522 nm (*λ*_ex_=455 nm), and *λ*_em_=516 nm (*λ*_ex_=417 nm), respectively. As a comparison, the fluorescence spectrum of **St8⋅**HCl was recorded showing similar behavior to those of the **3HBA** and **4HBA**‐based assemblies (*λ*_ex_=413 nm *λ*_em_=508 nm, for details see Figure S25). The significant hypsochromic shift of the **2HBA** system with respect to the other investigated assemblies can be attributed to the fact that hydrogen‐bonded complexes of **2HBA** emitting around 450 nm.^[23]^

Subsequently, the photo‐switchability of the fluorescence was investigated in detail. As **3HBA**(**St8**)_2_ performed best with respect to liquid‐crystalline properties and photo‐switchable fluorescence, we will focus our discussion on these materials. The **3HBA**(**St8**)_2_ assembly was brought into its nematic phase by heating to 130 °C. Upon irradiation with light (*λ*=405 nm) under the POM, the assemblies start to crystallize, and green fluorescence occurred (Figure S11). This result is in line with our previous observation and attributed to the crystallization of the stilbazolium salt upon photo‐initiated proton transfer, which is supported by the changes in the IR spectra (Figure S4). The band related to the hydrogen bond at around 2600 cm^−1^ undergoes a bathochromic shift of around 200 cm^−1^; additionally the C=O signal at ≈1690 cm^−1^ disappears. It should be noted that irradiation of the assembly in its crystalline state at room temperature did not affect the fluorescence, thereby proving the relevance of the dynamics in the liquid‐crystalline state for the performance of the material. In order to compare this behavior, fluorescence spectra of the **3HBA**(**St8**)_2_ assembly were recorded after different times of exposure to UV light (Figure 3). The spectra clearly show a tremendous increase in the fluorescence after irradiation of 10 s. Further irradiation did not result in significant changes of the fluorescence intensity. In addition, a slight shift in the maximum of emission wavelength was detected (from 530 to 522 nm).

**Figure 3:**
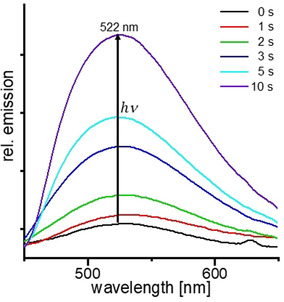
Fluorescence intensity (*λ*_ex_=420 nm) of crystalline **3HBA**(**St8**)_2_ samples at room temperature, after irradiation of the mesophase at 130 °C for 0 to 10 s with 365 nm.

It should be noted that the irradiated material does not show liquid‐crystalline behavior as supported by DSC data (Figure S22). However, resetting the samples to their initial nonfluorescent state was possible by heating the samples to 150 °C. After approximately 2 h the fluorescence completely vanished and the enantiotropic liquid‐crystalline behavior was recovered (Figure S22 and S24).

In order to prove the application potential of the photoswitchable fluorescence a thin film of **3HBA**(**St8**)_2_ was prepared and used for photo‐imprinting. Therefore, the film was partly covered by a photo mask showing the acronym of the University of Duisburg–Essen (UDE), heated to its nematic phase at 130 °C and irradiated with light of a wavelength of *λ*=405 nm for approximately 5 s. The sample was cooled to room temperature, and the mask was removed. Subsequently the film was observed under UV light. Although the covered regions remained dark, the irradiated areas of the films appeared highly fluorescent, due to photo‐initiated proton transfer (Figure 4 b). By heating the sample for 2 h to 150 °C the image was erased, and the film could be used for a second writing cycle.

**Figure 4:**
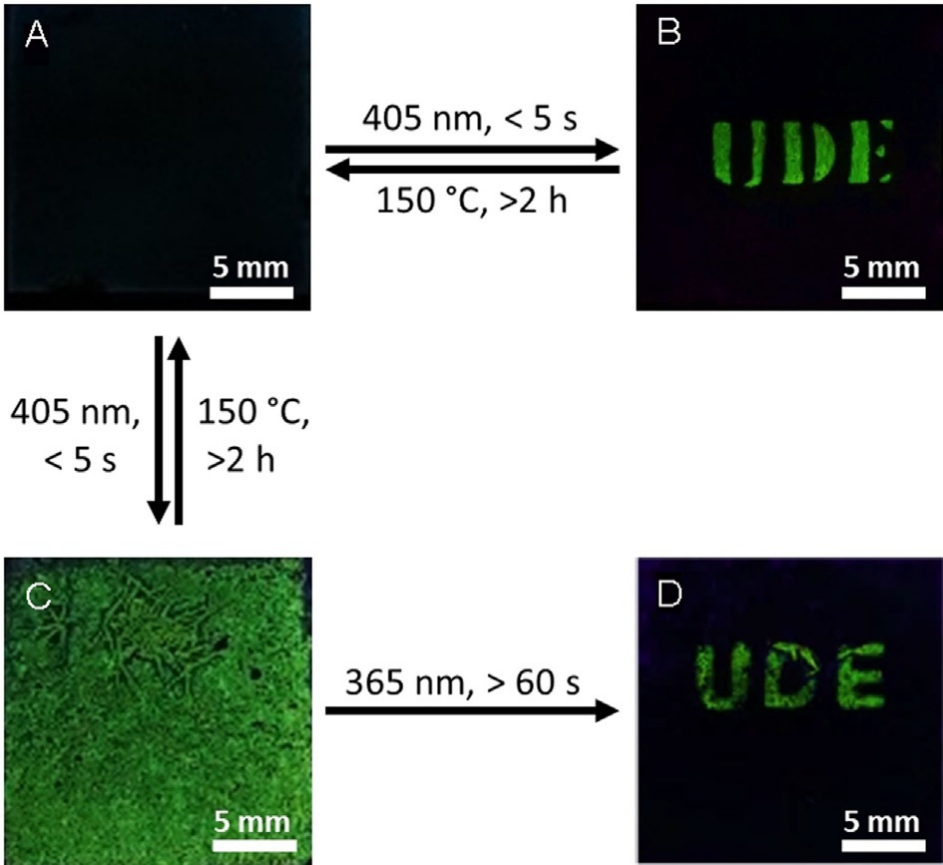
Behavior of **3HBA**(**St8**)_2_ samples under 405 nm light at room temperature, A) untreated sample, B) areas exposed to 405 nm light at 130 °C become fluorescent, C) whole sample exposed to 405 nm light at 130 °C, D) on uncovered areas the fluorescence is erased by 365 nm light at 25 °C.

In addition, we found that longer exposure times with 365 nm light yielded an irreversible photo‐pattern, due to the [2+2] cycloaddition of the excited stilbazole.[^22^, ^24^] This behavior was used for inverse photo‐patterning of the films. The whole sample was irradiated with 405 nm light for 5 s to yield a green fluorescent film (Figure 4 c). Partial covering of the films and subsequent irradiation with UV light (*λ*=365 nm) for 60 s yielded photo‐patterned films again showing the UDE acronym. The uncovered regions of the films lost their fluorescence due to [2+2] cycloaddition of the excited stilbazole, which could not be reversed under the chosen conditions. However, the fluorescence of the acronym could reversibly be switched on and off according to the previously described photo‐initiated proton transfer.

In conclusion, we have reported a supramolecular approach towards hydrogen‐bonded assemblies with photoswitchable fluorescence that results from a combination of hydrogen bonding, liquid crystallinity, and photobasicity. The hydrogen bonding between hydroxybenzoic acids and a stilbazole induces liquid crystallinity, which is crucial for the reversible, photoswitchable fluorescence of these materials. Irradiation of these materials in their mesophase with light (*λ*=405 nm) causes a photo‐initiated proton transfer from the hydrogen‐bond donor to the acceptor and turns on the fluorescence. This process can be used for reversible photo‐patterning of thin films by lithography. The study proves the relevance of a deep understanding of the complex interplay of noncovalent forces in supramolecular materials, which allows the materials’ properties to be controlled. We believe that the modularity of the chosen approach makes it appealing for the design of a variety of new supramolecular materials with switchable luminescence.

## Conflict of interest

The authors declare no conflict of interest.

## Supporting information

As a service to our authors and readers, this journal provides supporting information supplied by the authors. Such materials are peer reviewed and may be re‐organized for online delivery, but are not copy‐edited or typeset. Technical support issues arising from supporting information (other than missing files) should be addressed to the authors.

Supplementary
