# Supplementary material for: Photo‐switchable Fluorescence in Hydrogen‐Bonded Liquid Crystals
Source: Chemistry. 2020 Sep 17;26(59):13347–51. doi: 10.1002/chem.202001696 (PMC7693191; doi:10.1002/chem.202001696)
Supplement: Supplementary file 1 — Supplementary [file CHEM-26-13347-s001.pdf]

Chemistry–A European Journal

Supporting Information

Photo-switchable Fluorescence in Hydrogen-Bonded Liquid Crystals

Alexander Kappelt and Michael Giese*[a]

Switchable Fluorescence in Hydrogen-bonded Liquid Crystals

Alexander Kappelt[a] and Michael Giese* [a]

[a] Organic Chemistry, University Duisburg-Essen, Universitätsstraße 7, 45141 Essen, Germany. E-mail: michael.giese@uni-due.de.

Table of contents

1. Materials and Methods

2. Synthetic protocols

3. Characterisation of liquid crystalline complexes

4. Supplementary literature

1. Materials and Methods

Commercially available compounds and solvents were used as received from suppliers without further purification. 2-, 3-, and 4-hydroxy benzoic acid were obtained as ReagentPlus (99%) from Sigma Aldrich and were used without further purification. 1H-NMR-Spectra of the compounds were recorded in deuterated solvents (CDCl3 or DMSO-d6) with a Bruker DRX 300. Mass spectra were obtained with a Bruker amaZon (MS) and IR-spectra were recorded with a Bruker Alpha Platinum ATR IR-spectrometer. Polarized optical microscopy (POM) images were taken on a Nikon Eclipse Ni microscope with crossed polarizers equipped with a Linkam LTS420 heating stage and were recorded with an OpitixCam OCS-D3K2-14. For the photo responsive behaviour, for 405 nm a Laserpointer was used with 5 mW and for 365 nm a Hönle bluepoint LED eco with max. 10 W/cm2 was used normally with 15 % power. DSC thermograms were received using a DSC 3+/700/866/Argon from Mettler Toledo with a heating/cooling speed of 10°C/min (sample weight ~5 mg). Fluorescence measurements was performed with a Shimadzu RF-6000. CHNO analysis was performed on a EURO VECTOR EURO EA Elemental Analyzer.

2. Synthetic Protocols

2.1 Synthesis of (E)-4-(4-(octyloxy)styryl)pyridine (St-8)

The Synthesis was performed like described in literature[1].

^{1}H NMR (300 MHz, $CDCl_3$) δ = 8.54 (dd, *J* = 4.6, 1.6 Hz, 2H), 7.55 – 7.39 (m, 2H), 7.34 (dd, *J* = 4.8, 1.5 Hz, 2H), 7.26 (d, *J* = 16.3 Hz, 1H), 6.97 – 6.81 (m, 3H), 3.98 (t, *J* = 6.6 Hz, 2H), 1.89 – 1.57 (m, 2H), 1.55 – 1.21 (m, 8H), 0.90 (t, *J* = 6.8 Hz, 3H).

2.2 CHNO analysis

Table S1: *Comparison of the elemental analysis results with the calculated values.*

assembly	calc. C (%)	calc. H (%)	calc. N (%)	mes. C (%)	mes. H (%)	mes. N (%)
2HBA(St8)	75.14	7.43	3.13	75.5	7.45	2.685
2HBA$(St8)_2$	77.74	7.99	3.7	78.2	7.98	3.325
3HBA(St8)	75.14	7.43	3.13	75.4	7.34	2.87
3HBA$(St8)_2$	77.74	7.99	3.7	78.2	7.92	3.465
4HBA(St8)	75.14	7.43	3.13	75.6	7.39	2.78
4HBA$(St8)_2$	77.74	7.99	3.7	78.1	7.935	3.485

2.3 Synthesis and analysis of the supramolecular assemblies

The assemblies were obtained by dissolving the **2HBA**, **3HBA** or **4HBA** core moieties (1.0 eq.) and the side chain **St8** in the (1.0/2.0 eq.) separately in acetone. The solutions were subsequently combined and the solvent was removed under reduced pressure at 40°C, all under exclusion of light. All samples were grinded and dried in vakuum.

3. Characterization of liquid crystalline complexes

3.1 IR Spectra

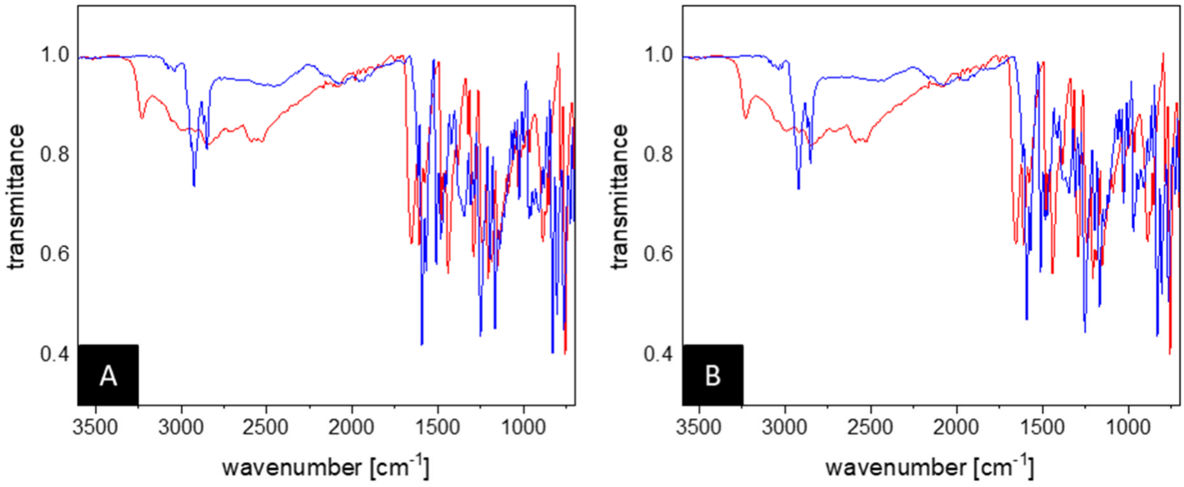

Figure S1. *IR spectra of **2HBA(St8)** (A) and **2HBA(St8)**$_2$ (B) in blue compared to **2HBA** in red.*

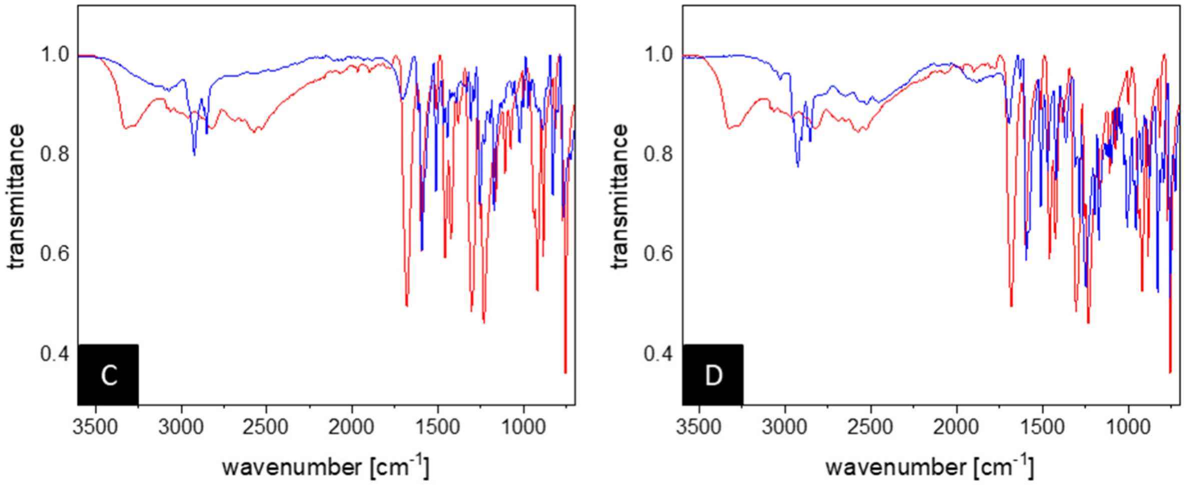

Figure S2. *IR spectra of **3HBA(St8)** (C) and **3HBA(St8)**$_2$ (D) in blue compared to **3HBA** in red.*

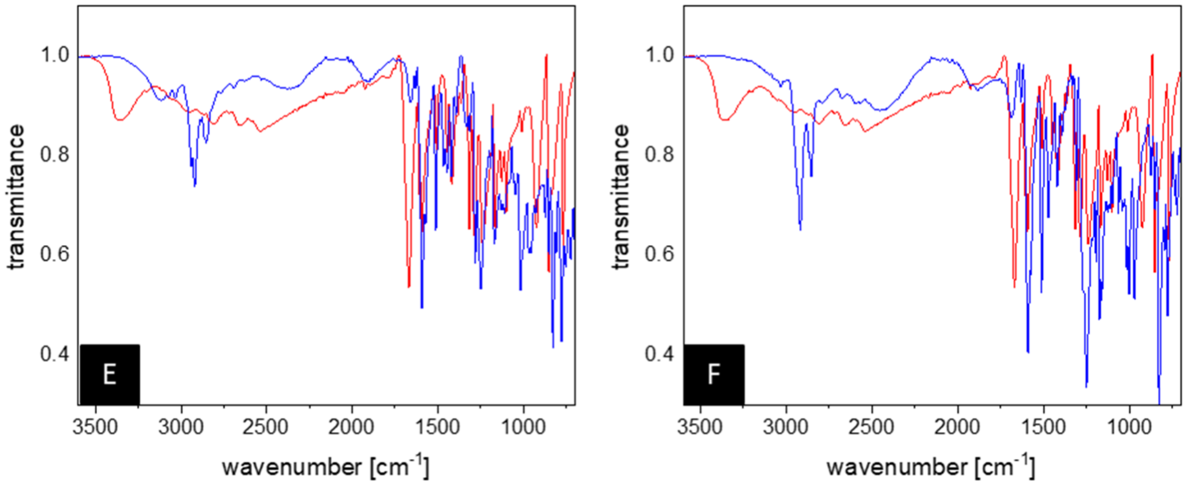

Figure S3. *IR spectra of **4HBA(St8)** (E) and **4HBA(St8)**$_2$ (F) in blue compared to **4HBA** in red.*

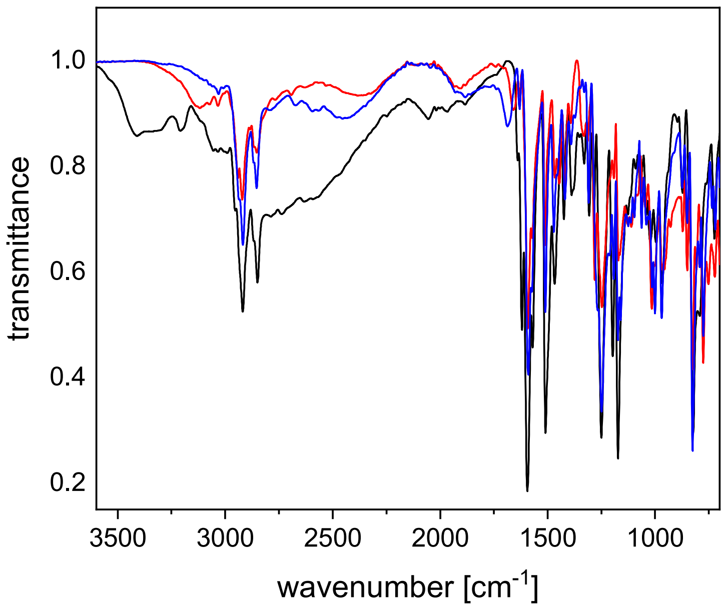

Figure S4. *IR spectra of untreated **4HBA(St8)**$_2$ (red), **4HBA(St8)**$_2$ after exposure to 405 nm (blue) and **St8**·HCl salt (black). The N-H band shifts from ~ 2600 cm^{-1} for the untreated sample to ~ 2470 cm^{-1} for the sample exposed to 405 nm, additionally the C=O signal at 1690 cm^{-1} disappears, which corresponds to the behavior carboxylic acids upon deprotonation, here the C=O band around 1700 cm^{-1} is replaced by one around 1600 cm^{-1}.*

3.2 POM-Images

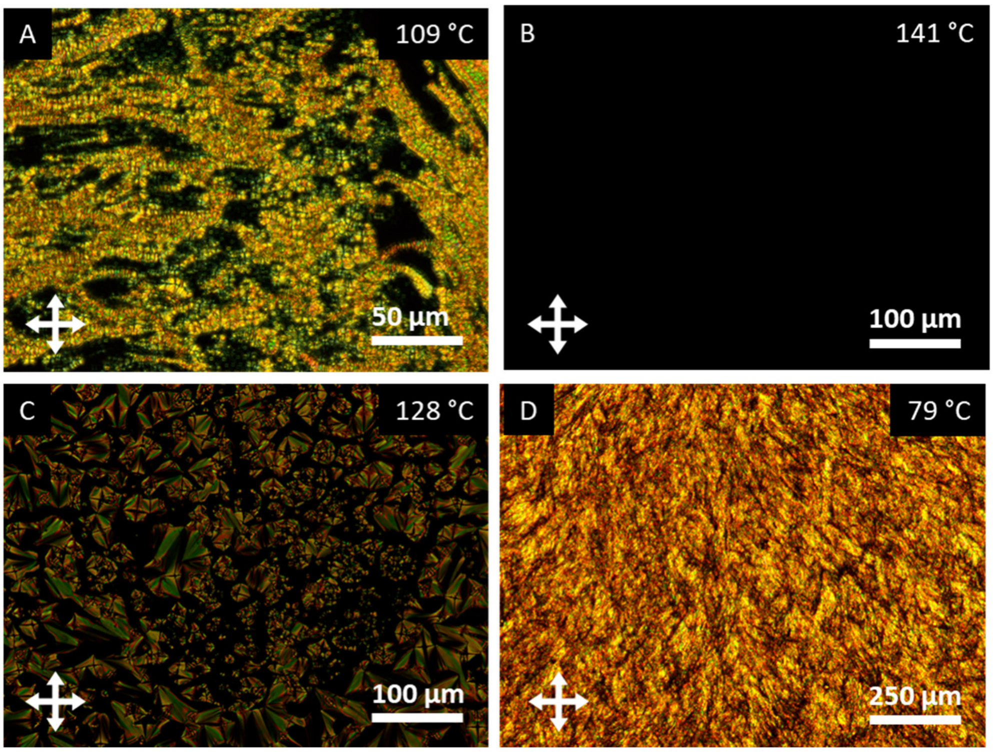

Figure S5. POM-Images of **2HBA**(**St8**) under crossed polarizer showing (A) a smectic phase at 109 °C upon heating, (B) isotropic at 141 °C, (C) a smectic phase at 128 °C upon cooling and (D) crystalline at 79 °C.

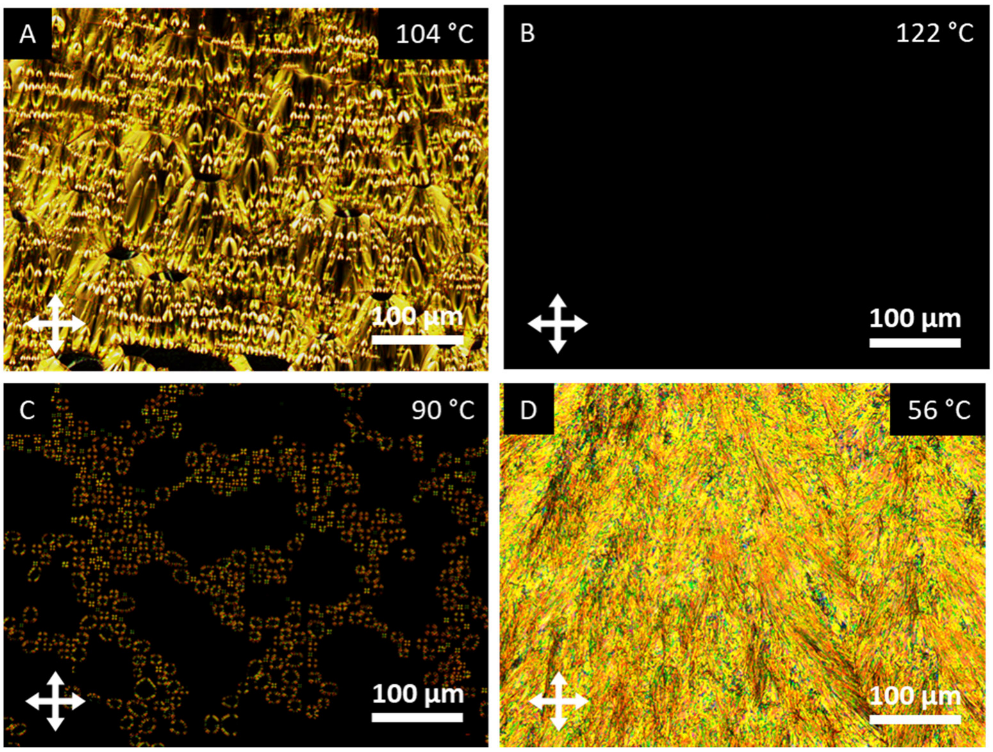

Figure S6. POM-Images of **2HBA**(**St8**)$_2$ under crossed polarizer showing (A) a smectic phase at 104 °C upon heating, (B) isotropic at 122 °C, (C) a smectic phase at 90 °C upon cooling and (D) crystalline at 56 °C.

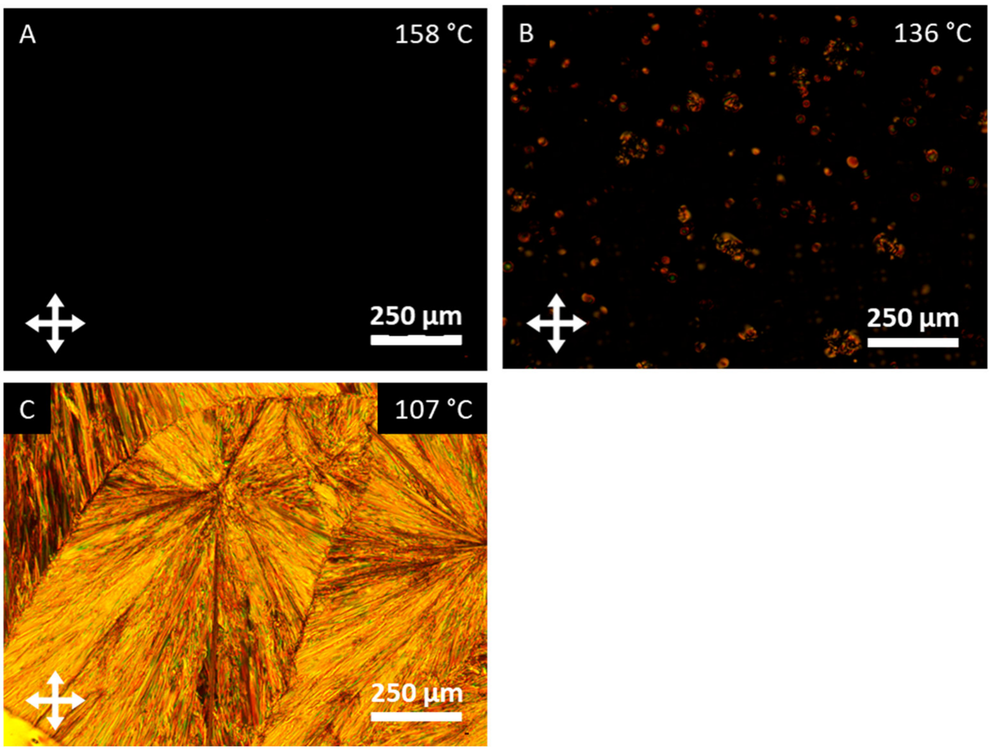

Figure S7. POM-Images of **3HBA**(**St8**) under crossed polarizer showing (A) isotropic phase at 158 °C upon heating, (B) nematic phase at 136 °C and (C) crystalline at 107 °C.

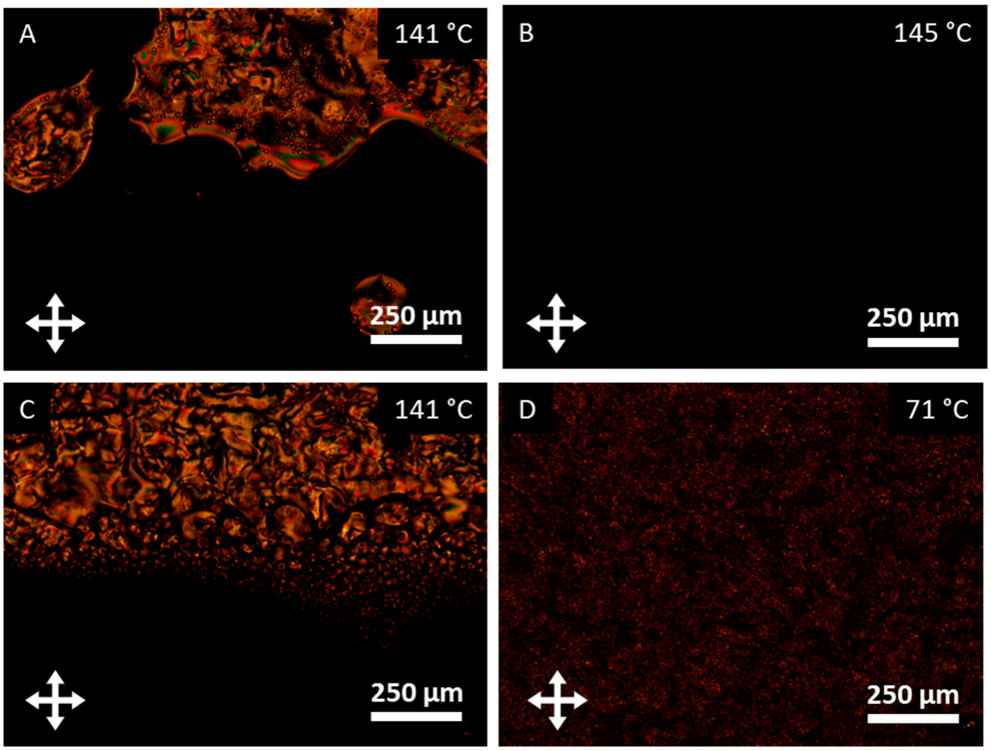

Figure S8. POM-Images of **3HBA**(**St8**)$_2$ under crossed polarizer showing (A) a nematic phase at 141 °C upon heating, (B) isotropic at 145 °C, (C) a nematic phase at 128 °C upon cooling and (D) crystalline at 71 °C.

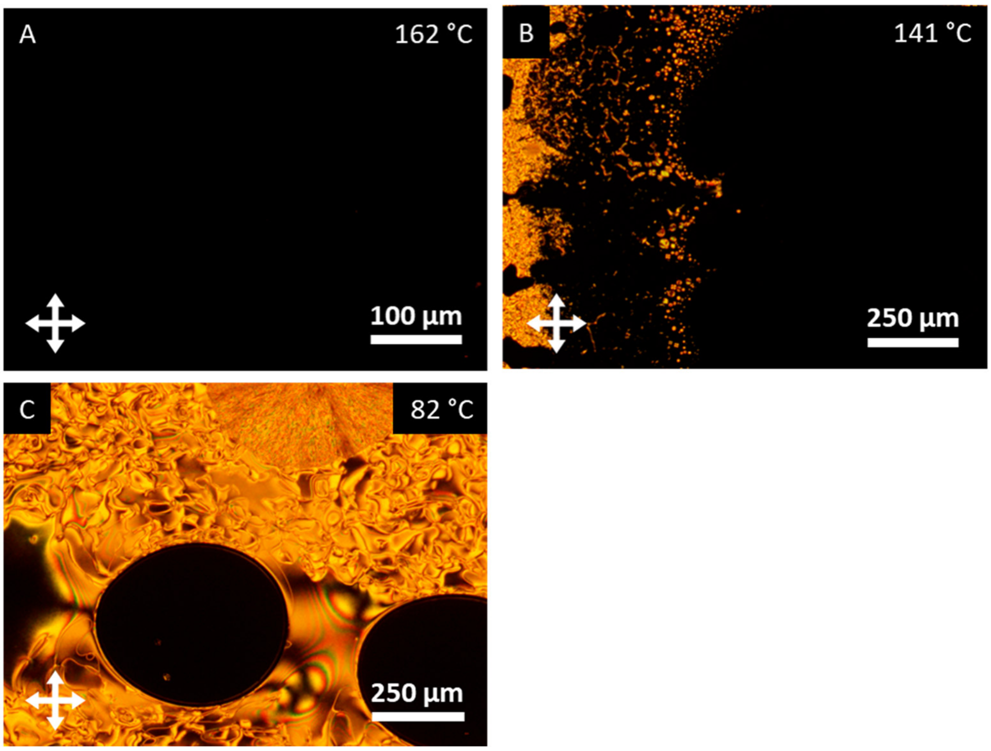

Figure S9. POM-Images of **4HBA**(**St8**) under crossed polarizer showing (A) isotropic phase at 162 °C upon heating, (B) a nematic phase at 141 °C upon cooling and (D) crystallization at 82 °C.

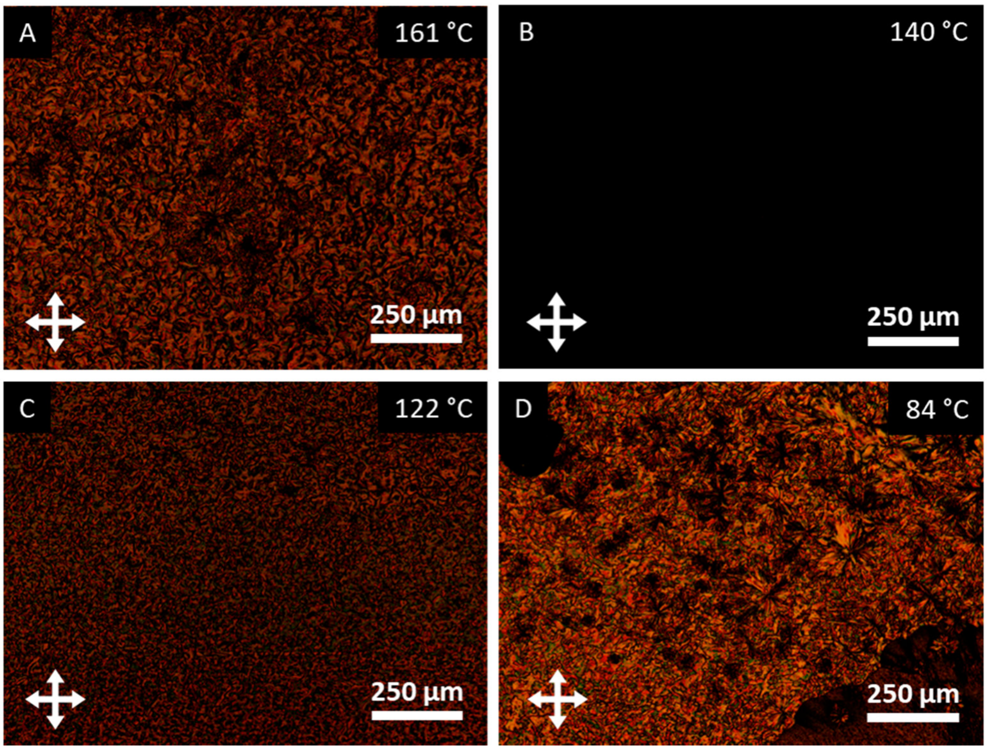

Figure S10. POM-Images of **4HBA**(**St8**)$_2$ under crossed polarizer showing (A) a nematic phase parallel to crystalline domains at 115 °C upon heating, (B) isotropic at 161 °C, (C) a nematic phase at 122 °C upon cooling and (D) crystallization at 84 °C.

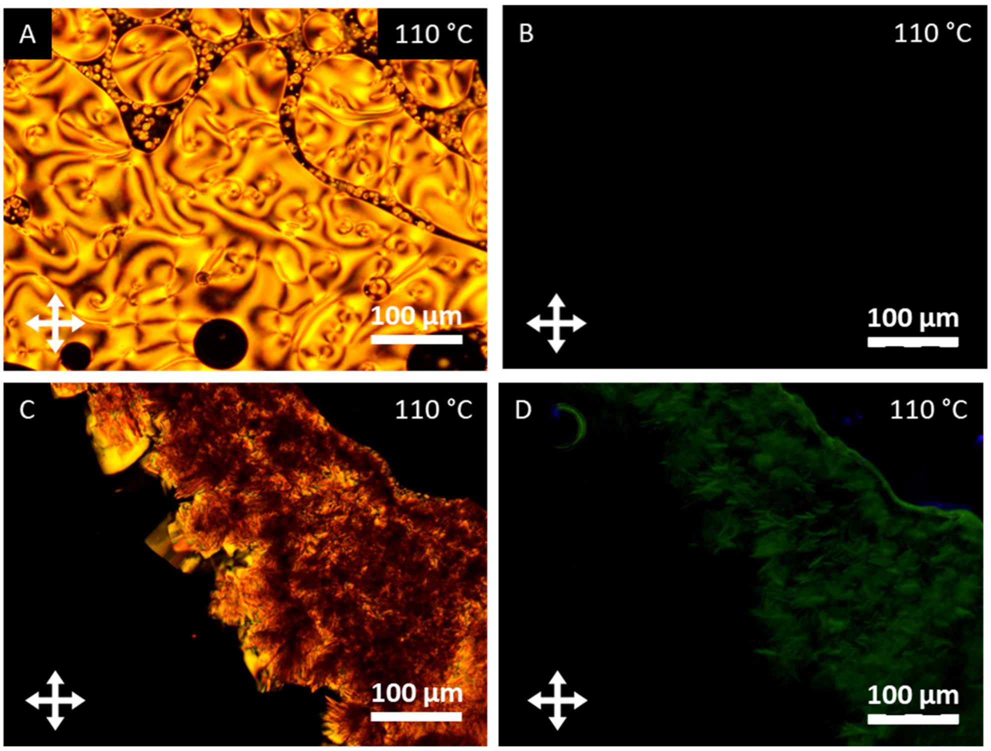

Figure S11. POM-Images of **4HBA**(**St8**)$_2$ under crossed polarizer at 110 °C showing (A) a untreated nematic phase, (B) isotropic phase short after irradiation with 405 nm, (C) starting crystallization after prolonged exposure to 405 nm and (D) fluorescence of the formed crystals.

3.3 DSC Data

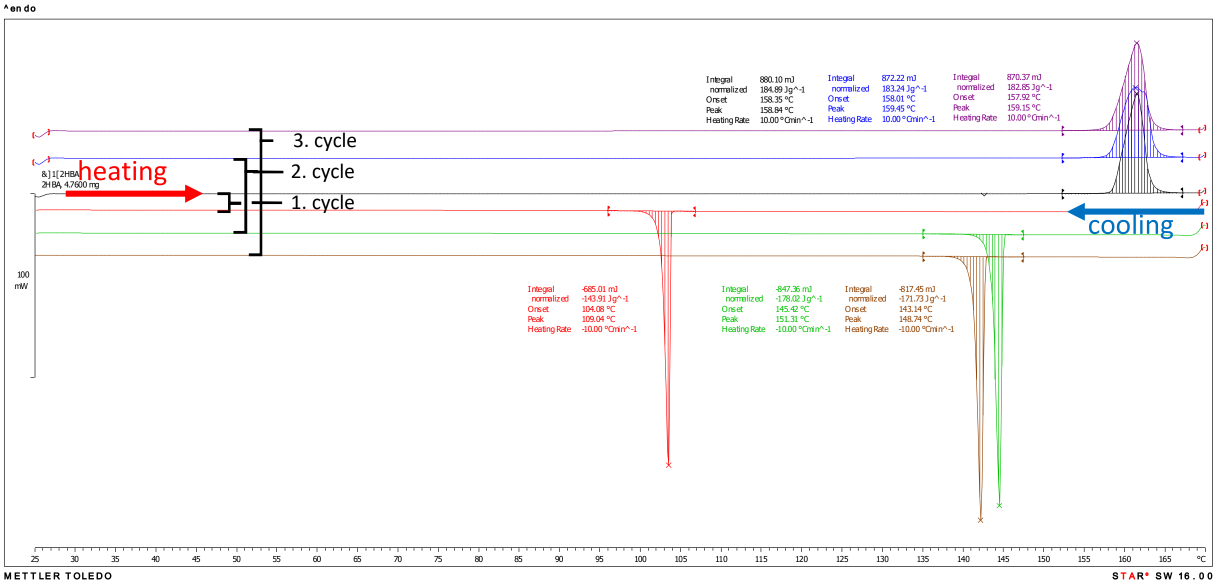

Figure S12. *DSC traces of* ***2HBA*** *measured with 10 $^{K}/_{min}$.*

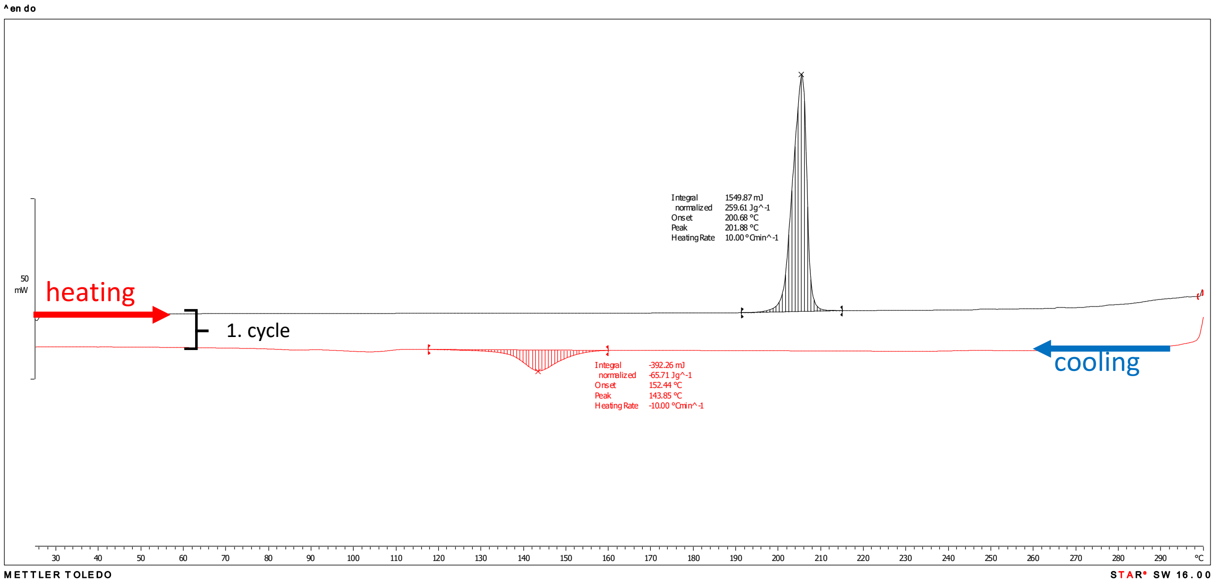

Figure S13. *DSC traces of* ***3HBA*** *measured with 10 $^{K}/_{min}$.*

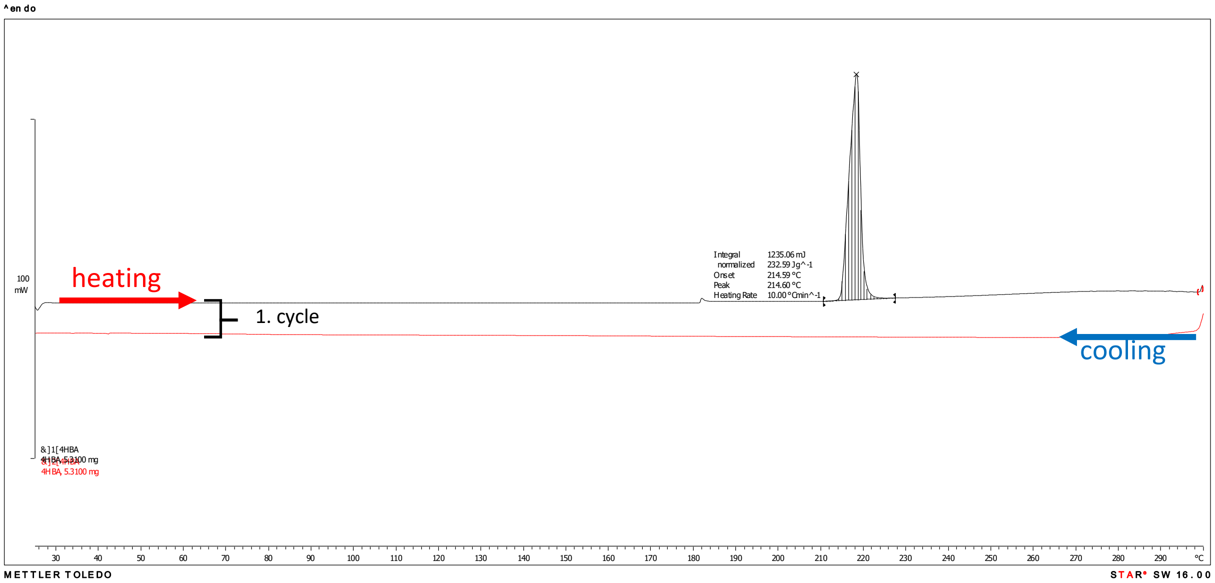

Figure S14. *DSC traces of **4HBA** measured with 10 $^{K}/_{min}$.*

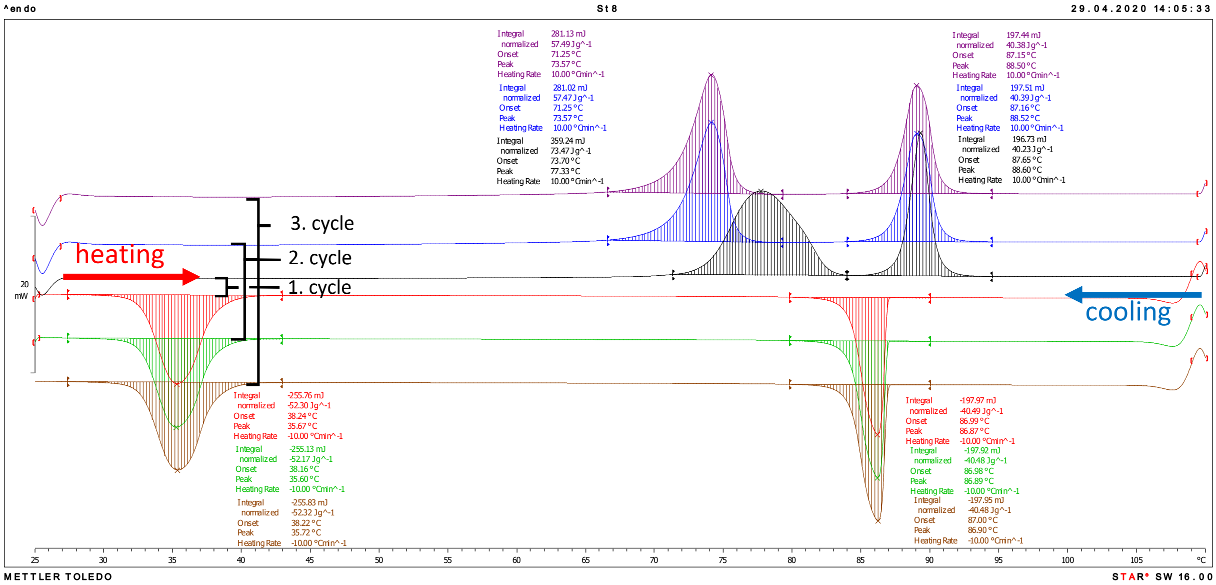

Figure S15. *DSC traces of **St8** measured with 10 $^{K}/_{min}$.*

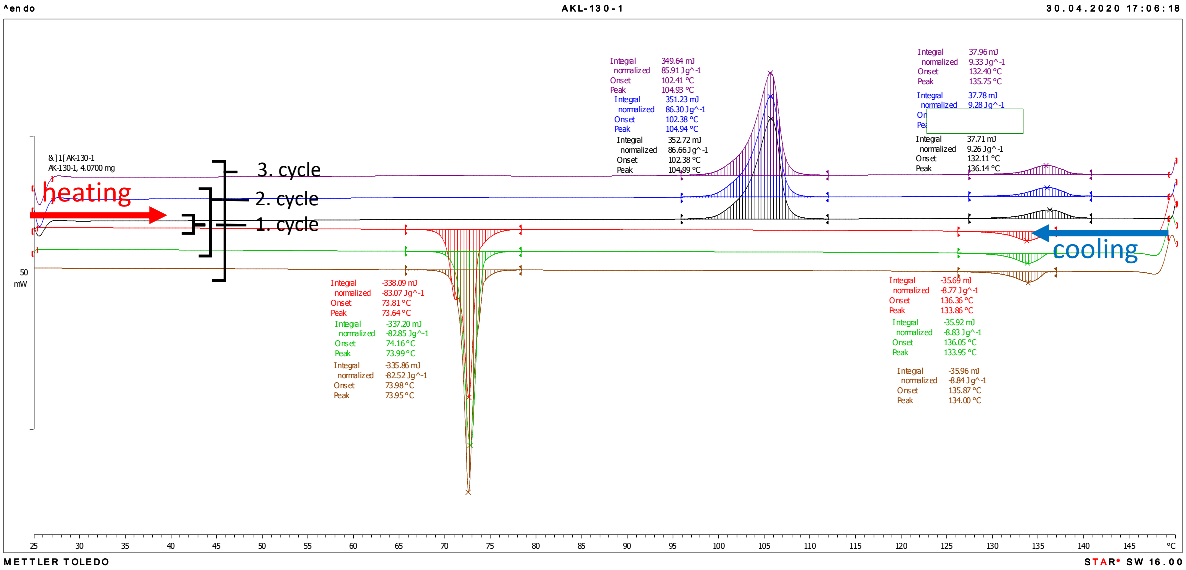

Figure S16. *DSC traces of **2HBA(St8)** measured with 10 $^{K}/_{min}$.*

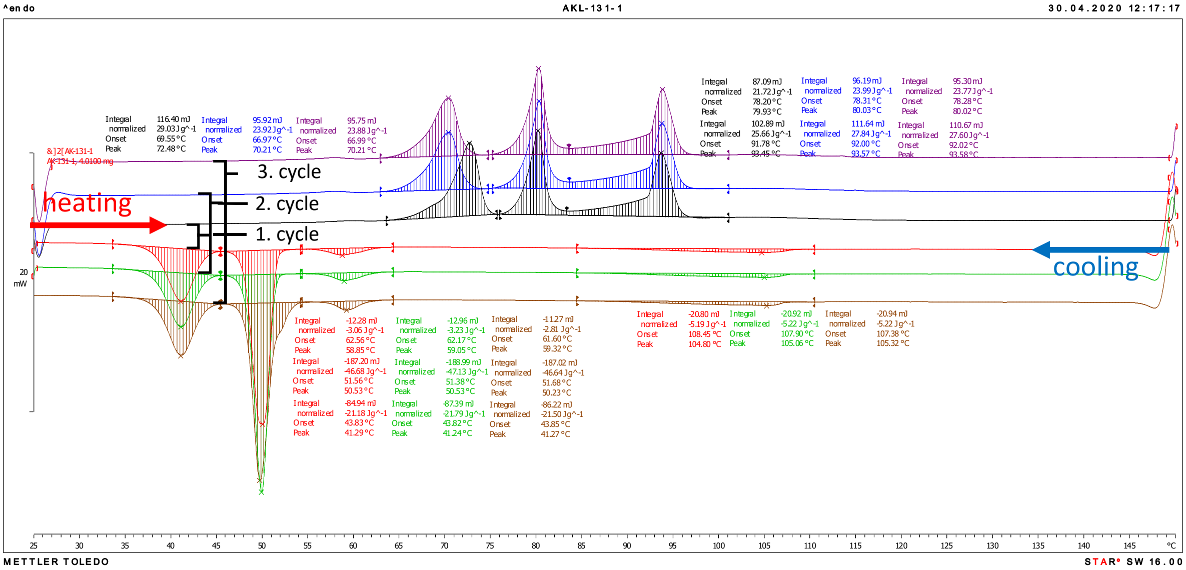

Figure S17. *DSC traces of **2HBA(St8)**$_2$ measured with 10 $^{K}/_{min}$.*

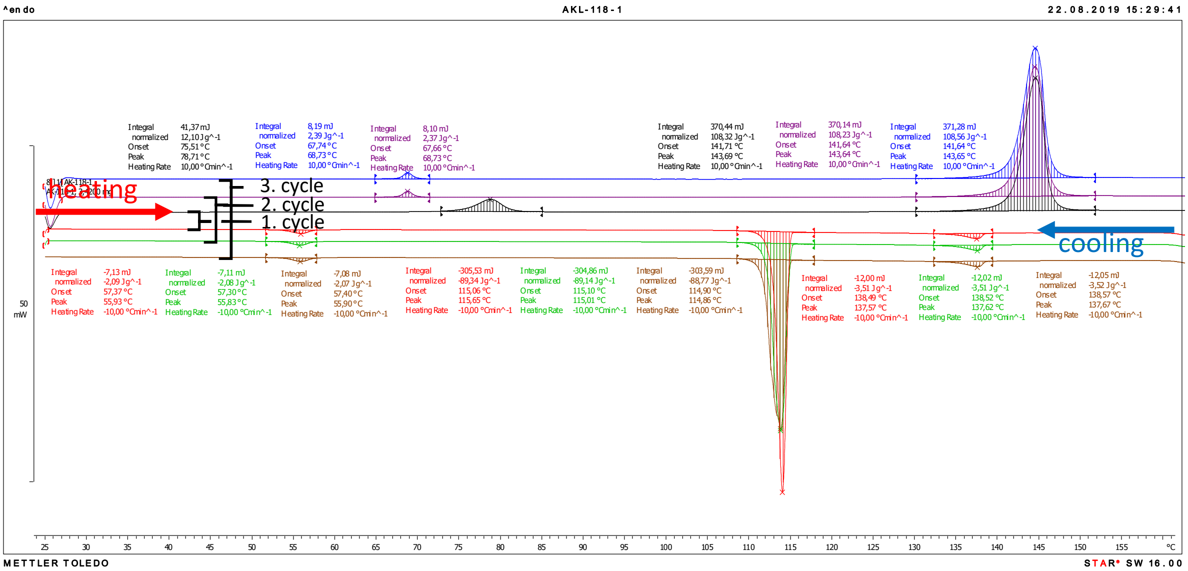

Figure S18. *DSC traces of **3HBA(St8)** measured with 10 $^{K}/_{min}$.*

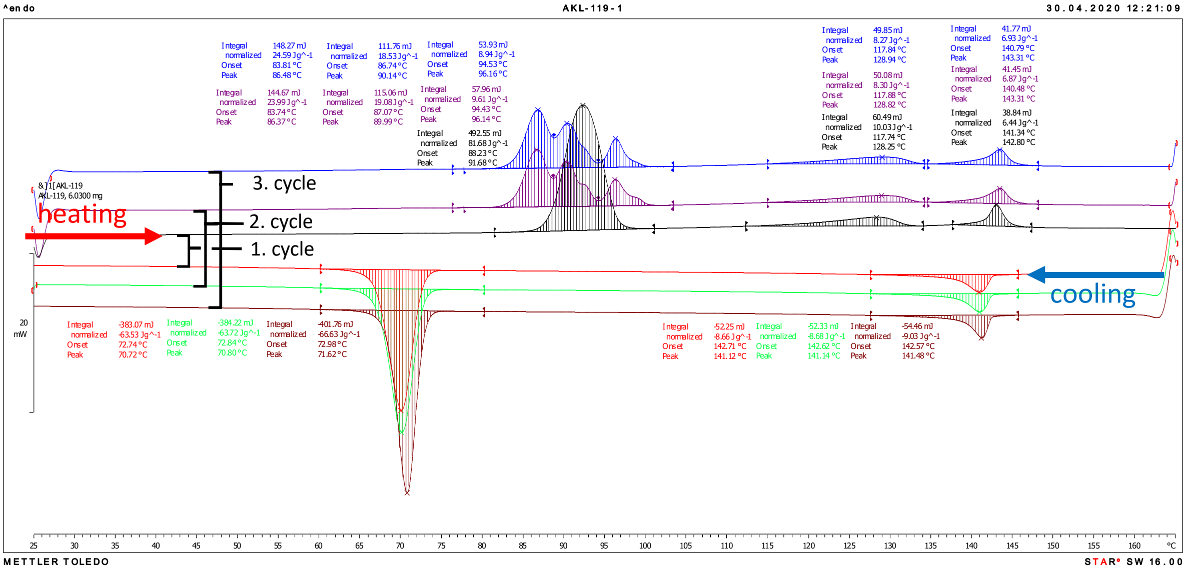

Figure S19. *DSC traces of **3HBA(St8)**$_2$ measured with 10 $^{K}/_{min}$.*

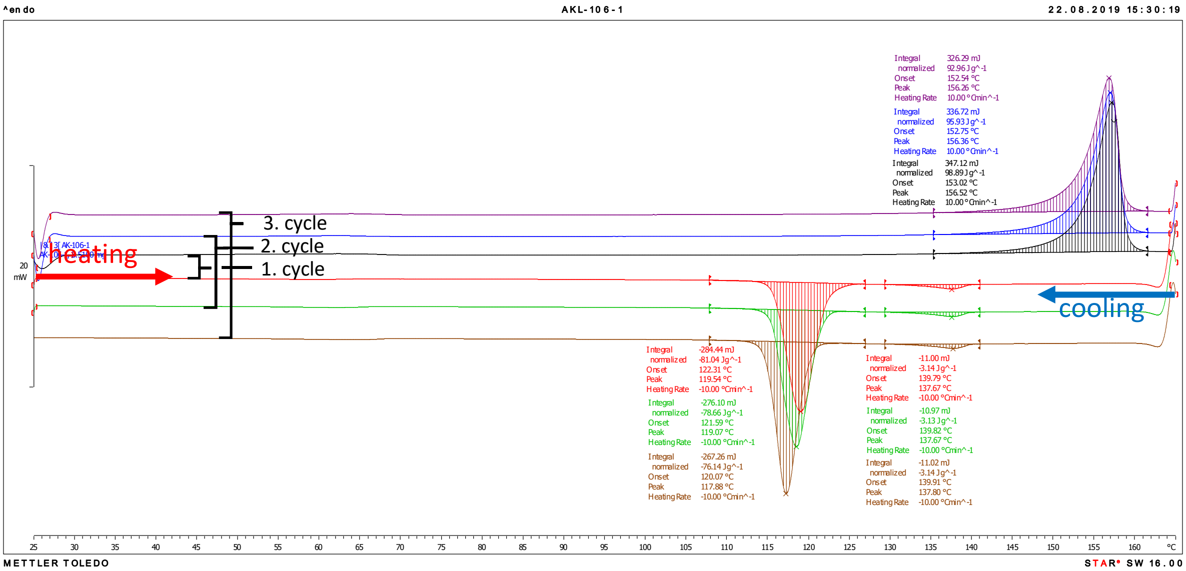

Figure S20. *DSC traces of* ***4HBA(St8)*** *measured with 10* $^{K}/_{min}$.

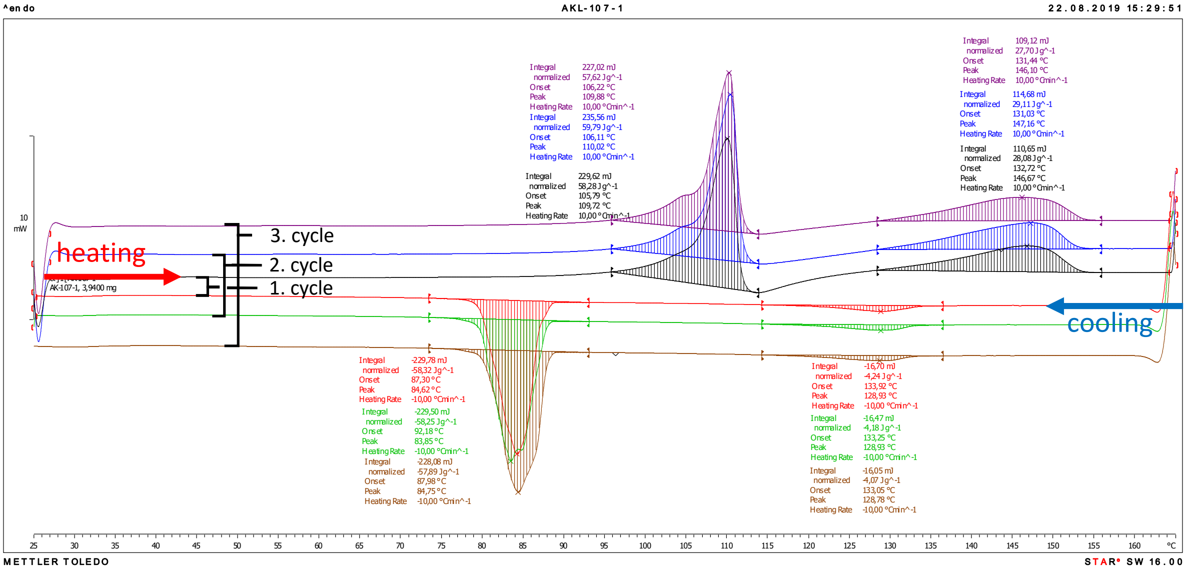

Figure S21. *DSC traces of* ***4HBA(St8)***$_2$ *measured with 10* $^{K}/_{min}$.

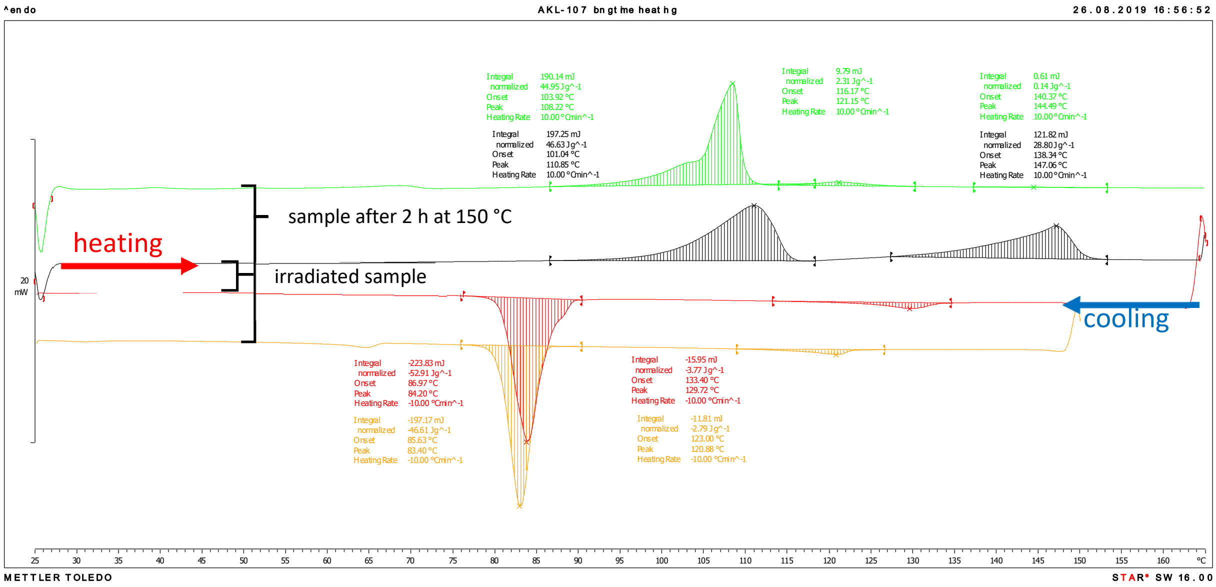

Figure S22. *DSC traces of **4HBA(St8)**$_2$ treaded with 405 nm at 130 °C for 15 s (black/red) and after 2 h isothermic at 150 °C (green/yellow) measured with 10 $^{k}/_{min}$.*

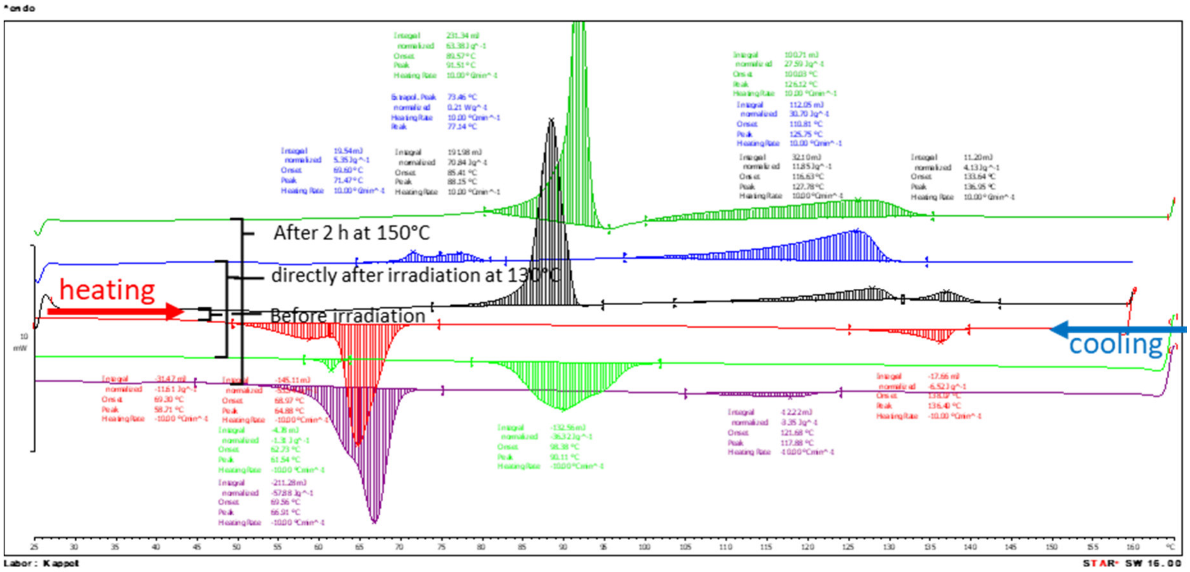

Figure S23. *DSC traces of **3HBA(St8)**$_2$ untreated (black/red), irradiated with 405 nm at 130 °C (blue/green) and after 2h heating at 150 °C (green/violet) measured with 10 $^{k}/_{min}$.*

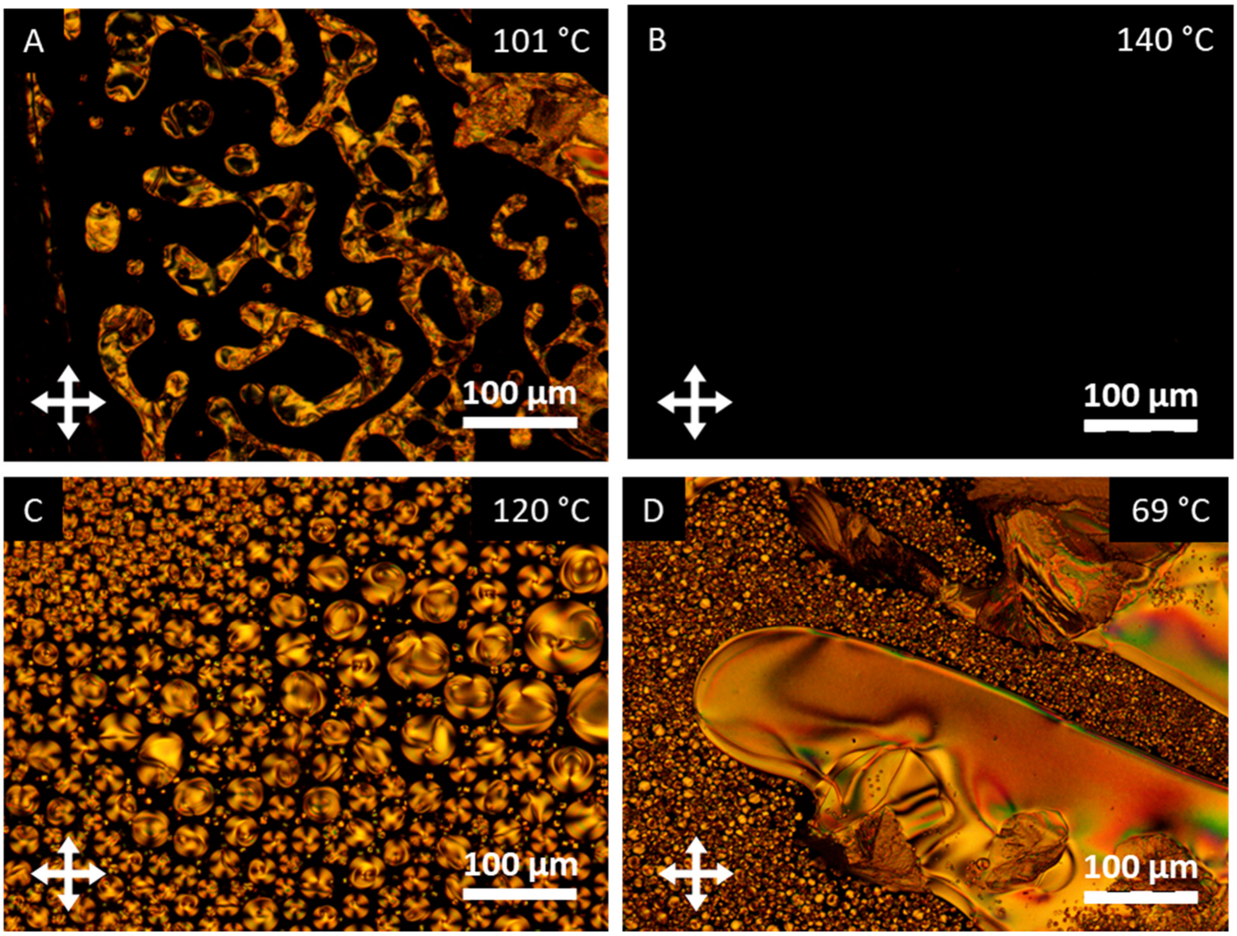

Figure S24. POM-Images of **3HBA**(**St8**)$_2$, after irradiation and reset at 150 °C for 18 h, under crossed polarizer showing (A) a nematic phase at 101 °C upon heating, (B) isotropic at 140 °C, (C) a nematic phase at 120 °C upon cooling and (D) crystallization at 69 °C.

Table S2: Summary of thermal properties. [a] decomposition.

assembly	Thermal data								
		T (°C)	H (J·g^{-1})		T (°C)	H (J·g^{-1})		T (°C)	H (J·g^{-1})
**2HBA**(**St8**)	Cr→Sm	104.94	86.30	Sm→I	135.75	9.33	-	-	-
	I→Sm	133.95	-8.83	Sm→Cr	73.99	-82.52	-	-	-
**2HBA**(**St8**)$_2$	Cr1→Cr2	70.21	23.92	Cr2→Cr3	80.03	23.99	Cr3→→I	93.57	27.84
	I→Sm	105.06	-5.22	Sm→M	59.05	-3.23	M→Cr2	50.53	-47.13
	Cr2→Cr1	41.24	-21.79	-	-	-	-	-	-
**3HBA**(**St8**)	Cr1→Cr2	68.73	2.39	Cr2→I	143.64	108.23	Cr2→Cr1	55.83	-2.08
	I→N	137.62	-3.51	N→Cr2	115.01	-89.14			
**3HBA**(**St8**)$_2$	Cr1→Cr2	86.37	23.99	Cr2→Cr3	89.99	19.08	Cr3→Cr4	96.14	9.61
	Cr4→N	128.82	8.30	N → I	143.31	6.44	-	-	-
	I→N	141.48	-9.03	N→Cr1	71.62	-66.63			
**3HBA**(**St8**)$_2$	Cr1→Cr2	71.47	5.35	Cr2→N	127.78	30.70	-	-	-
irradiated	N→Cr2	90.11	-36.32	Cr2→Cr1	61.45	-1.31	-	-	-
**4HBA**(**St8**)	Cr→I	156.52	95.93	I→N	137.67	-10.97	-	-	-
	N→Cr	119.07	-78.66	-	-	-	-	-	-
**4HBA**(**St8**)$_2$	Cr1→Cr2	106.11	57.62	Cr2→I	147.16	29.11	I→N	128.93	-4.18
	I→Cr1	83.85	-58.25	-	-	-	-	-	-
**4HBA**(**St8**)$_2$	Cr1→Cr2	110.85	46.63	Cr2→I	147.06	28.80	-	-	-
irradiated	I→N	129.72	-3.77	N→Cr1	86.97	-52.91	-	-	-
**4HBA**(**St8**)$_2$	Cr1→N	108.22	44.95	N→I	121.15	2.31	-	-	-
reseted	I→N	120.88	-2.79	N→Cr1	83.40	-46.61	-	-	-
**2HBA**	Cr→I	149.45	183.24	-	-	-	-	-	-
	I→Cr	141.31	-178.02	-	-	-	-	-	-
	Cr→I	201.88 [a]	259.61	-	-	-	-	-	-

**3HBA**	I→Cr	152.44 [a]	-65.71	-	-	-	-	-	-
**4HBA**	Cr→I	214.60 [a]	232.59	-	-	-	-	-	-
	I→Cr			-	-	-	-	-	-
**St8**	Cr1→Cr2	73.57	57.47	Cr2→I	88.52	40.39	-	-	-
	I→Cr2	86.87	-40.49	Cr2→Cr1	35.60	-52.30	-	-	-

3.4 Fluorescence spectra

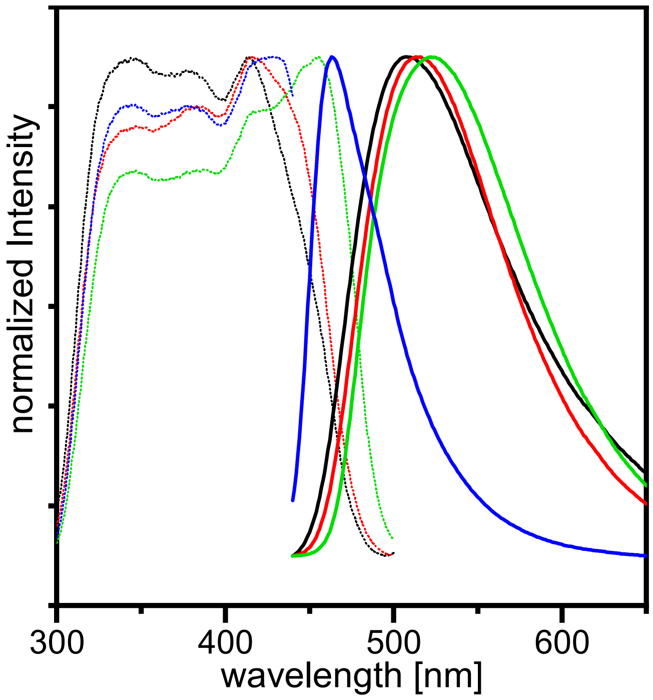

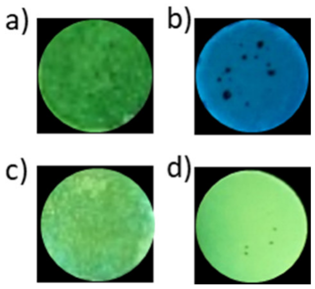

Figure S25 *(left) Comparison of the excitation (dotted) and emission (solid) spectra of* ***St8*****HCl (black),* ***2HBA(St8)*** *(blue),* ***3HBA(St8)***$_2$ *(green) and* ***4HBA(St8)***$_2$ *(red). (right) Pictures of the visible fluorescence under 405 nm light of a)* ***St8*****HCl, b)* ***2HBA(St8)****, c)* ***3HBA(St8)***$_2$ *and d)* ***4HBA(St8)***$_2$*.*

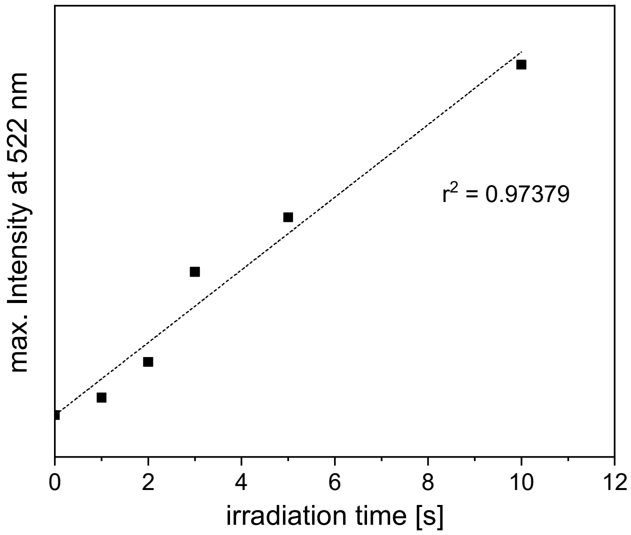

Figure S26 *Plot of the maxima shown in Fig 3. against the irradiation time.*

4. Supplementary literature

1. L. Cui and Y. Zhao, *Chem. Mater.*, 2004, **16**(11), 2076.
2. C. Koopmans and H. Ritter, *J. Am. Chem. Soc.*, 2007, **129**(12), 3502.
3. M. Giese, T. Krappitz, R. Y. Dong, C. A. Michal, W. Y. Hamad, B. O. Patrick and M. J. MacLachlan, *J. Mater. Chem. C*, 2015, **3**(7), 1537.
4. M. Saccone, M. Pfletscher, E. Dautzenberg, R. Y. Dong, C. A. Michal and M. Giese, *J. Mater. Chem. C*, 2019, **7**(11), 3150.
5. R. Cano, *Bulletin de la societe francaise mineralogie et de cristallographie*, 1967, **90**(3), 333.
